# Effect of 1.8 GHz radiofrequency electromagnetic radiation on novel object associative recognition memory in mice

**DOI:** 10.1038/srep44521

**Published:** 2017-03-17

**Authors:** Kai Wang, Jun-Mei Lu, Zhen-He Xing, Qian-Ru Zhao, Lin-Qi Hu, Lei Xue, Jie Zhang, Yan-Ai Mei

**Affiliations:** 1 State Key Laboratory of Medical Neurobiology, School of Life Sciences and Institutes of Brain Science, Fudan University, Shanghai, 200433, China; 2 Physics department, East China Normal University, Shanghai, 200241, China

## Abstract

Mounting evidence suggests that exposure to radiofrequency electromagnetic radiation (RF-EMR) can influence learning and memory in rodents. In this study, we examined the effects of single exposure to 1.8 GHz RF-EMR for 30 min on subsequent recognition memory in mice, using the novel object recognition task (NORT). RF-EMR exposure at an intensity of >2.2 W/kg specific absorption rate (SAR) power density induced a significant density-dependent increase in NORT index with no corresponding changes in spontaneous locomotor activity. RF-EMR exposure increased dendritic-spine density and length in hippocampal and prefrontal cortical neurons, as shown by Golgi staining. Whole-cell recordings in acute hippocampal and medial prefrontal cortical slices showed that RF-EMR exposure significantly altered the resting membrane potential and action potential frequency, and reduced the action potential half-width, threshold, and onset delay in pyramidal neurons. These results demonstrate that exposure to 1.8 GHz RF-EMR for 30 min can significantly increase recognition memory in mice, and can change dendritic-spine morphology and neuronal excitability in the hippocampus and prefrontal cortex. The SAR in this study (3.3 W/kg) was outside the range encountered in normal daily life, and its relevance as a potential therapeutic approach for disorders associated with recognition memory deficits remains to be clarified.

Radiofrequency (RF) fields are used extensively in wireless communications, including mobile phones, radio, and television. Previous *in vitro* and *in vivo* experimental studies have shown that RF electromagnetic radiation (RF-EMR) emitted from mobile phones can alter biological functions, such as the balance between oxidants, antioxidants, and neurotransmitters, as well as gene/protein expression and neuroinflammation^1^,^2^,^3^,^4^. Because RF fields are in close proximity to the brain during mobile-phone use, whether such exposure can result in brain dysfunction, particularly in terms of learning and memory, is a major topic of investigation^5^. However, previous studies have produced conflicting evidence regarding the effects of exposure to RF-EMR on learning and memory as a result of methodological differences in experimental setups, exposure durations, the animal models used, and the behavioral test(s) used to asses learning and/or memory.

Aldad *et al*. reported that exposure of fetal mice to RF radiation from 800–1900 MHz-rated cellular telephones (24 h/day throughout gestation) affected neuron development and subsequent behavior. Exposed subjects showed memory impairments, as determined by the novel object recognition task (NORT)^6^. In contrast, Daniels *et al*. reported that RF-EMR exposure had no effect on either spatial memory or brain morphology, but did decrease locomotor activity in rats^7^. Arendash *et al*. suggested that long-term, high-frequency RF-EMR exposure (918 MHz; 0.25 W/kg, 1 h/day for 4–7 months) protected against and even reversed cognitive impairment in a mouse model of Alzheimer’s disease^8^. However, it should be noted that these recent *in vitro* and *in vivo* studies used a relatively low RF-EMR density and long-term (chronic) exposure. The effects of single, high-power (1.8 GHz) RF-EMR exposure on rodent learning and memory thus remain to be established.

Both rats and mice tend to interact more with novel than with familiar objects. This natural tendency has been exploited in many behavioral assays, notably the NORT^9^. NORT is used by behavioral pharmacologists and neuroscientists to study learning and memory, including short-term, intermediate, and long-term memories. The test involves manipulating the retention interval, i.e., the length of time animals must remember the sample objects presented during the familiarization phase, but before the test phase, at which point one of the familiar objects is replaced by a novel one^10^. Previous studies have shown that the NORT is associated with various hippocampal functions^11^. Although the exact role of the hippocampus in object recognition memory remains under debate, apparent discrepancies in the literature may be largely associated with methodological variations, e.g., in type and age of study animals, object shape and color, and testing environment^10^. In a recent study, we found that exposure to low-frequency (50 Hz) electromagnetic fields (1 mT, 12 h/day) induced a time-dependent deficit in novel object associative recognition memory and decreased hippocampal dendritic-spine density, with no corresponding changes in spontaneous locomotor activity. Furthermore, this effect was both transient and reversible^12^. Chen *et al*. reported that 1.8 GHz RF-EMF could impair neurite outgrowth in embryonic neural stem cells NSCs *in vitro*^13^. Whether RF-EMR exposure also affects novel object associative recognition memory and hippocampal dendritic-spine density thus remains to be explored.

Several studies have reported that high frequency RF-EMR exposure can affect central nervous system functioning as well as learning and memory in rodent models, but the molecular and cellular effects of RF-EMR exposure have not yet been fully characterized. In addition to potentially activating extracellular signal-regulated kinase signal transduction pathways and affecting reactive oxygen species formation, resulting in possible cellular metabolic dysfunction and/or membrane dyspermeability via oxidative stress^14^,^15^,^16^, Wang *et al*. suggested that impairment of long-term potentiation induction and structural damage to the hippocampus both contributed to RF-EMR exposure-induced deficits in cognitive functions^17^. In addition to changing hippocampal morphology and/or functioning, other studies have demonstrated that prenatal exposure to mobile-phone radiation can cause adult memory impairment, as measured by NORT^6^. Such an effect has been suggested to result from impaired glutamatergic synaptic transmission to pyramidal cells in the prefrontal cortex, implying that cortical neurons are also associated with NORT and that their excitability may be affected by RF-EMR exposure^6^. The observation that cortical neuron morphology and activity may underlie the effects of RF-EMR exposure in mice may provide the basis for identifying the biological mechanisms responsible for the effects of RF-EMR exposure.

The present study aimed to determine the effects of single, high-density exposure to mobile-phone radiation (1.8 GHz) on novel object recognition memory, dendritic-spine morphology, and the excitability of pyramidal cells in the hippocampal and prefrontal cortices in mice, using behavioral tests (open field and object recognition tests), histology (Golgi staining), and electrophysiology (whole-cell current-clamp).

## Results

### RF-EMR exposure enhanced novel object recognition memory with no change in locomotor activity

**Figure 1:**
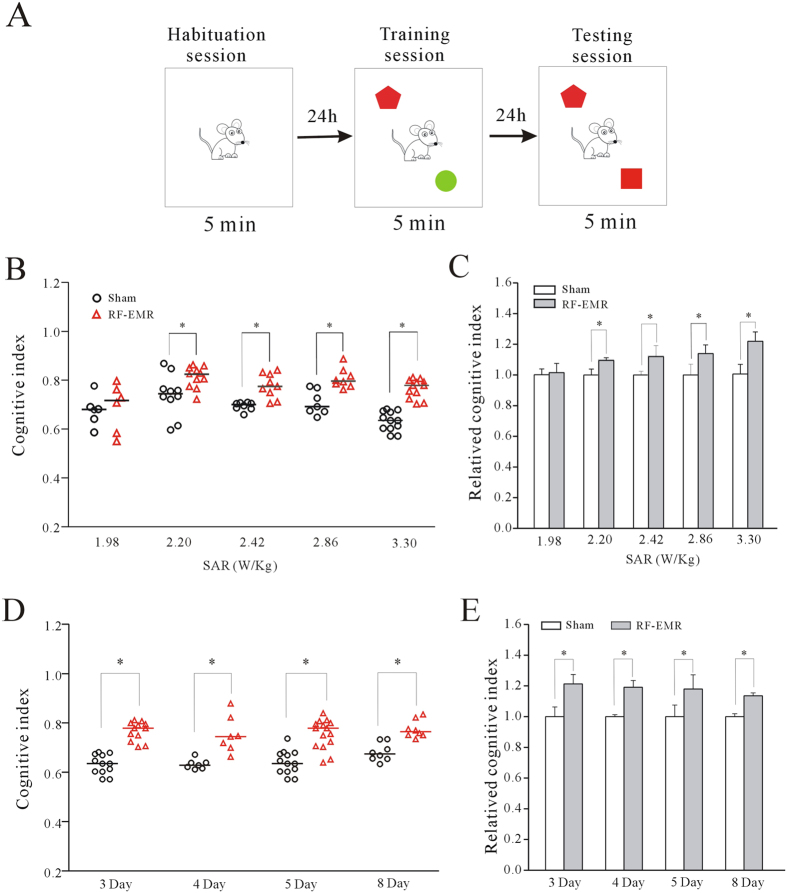
Effect of 1.8 GHz RF-EMR exposure on novel object recognition memory in mice detected using NORT. (**A**) Behavioral scheme for NORT training and testing. (**B**) Original data from exposed mice subjected to RF-EMR at a SAR of 1.98–3.30 W/kg for 30 min. *p < 0.05 for two groups connected by a straight line using a one-way Kruskal–Wallis test with Dunn’s post-hoc test. (**C**) Original data were normalized to their corresponding control to show the intensity-dependent influence on recognition index in mice. *p < 0.05 for two groups connected by a straight line using a two-sample *t*-test. (**D**) Original data present the effect of 3.30 W/kg RF-EMR exposure on recognition index in mice on different days after RF-EMR exposure. *p < 0.05 for two groups connected by a straight line using a one-way Kruskal–Wallis test with Dunn’s post-hoc test. (**E**) The data were normalized to non-RF-EMR-exposed (sham-exposed) mice on the same day. *p < 0.05 for two groups connected by a straight line using a two-sample *t*-est.

We used NORT to determine if RF-EMR exposure affected murine recognition memory. A schematic of the NORT procedure is shown in Fig. 1A. The ability of each group to recognize the novel object was determined by dividing the mean time spent exploring the novel object by the total mean time exploring both the novel and familiar objects during the test session. This value was multiplied by 100 to obtain a percentage preference for the novel object. This was termed the recognition index, according to the standard formula: (T_novel_/[T_novel_ + T_familiar_] × 100).

We then assessed the effect of RF-EMR exposure on long-term recognition memory by comparing the recognition indices between control (sham-exposed) and RF-EMR-exposed mice. The data obtained from mice exposed to RF-EMR at a specific absorption rate (SAR) of 1.98–3.30 W/kg for 30 min before the first trial are shown in Fig. 1B. NORT began on the third day after RF-EMR exposure. The original data were normalized to their corresponding control (non-RF-EMR exposure). There was no significant difference between sham-exposed and 1.98 W/kg RF-EMR-exposed mice, indicating that the increase in recognition was SAR-dependent (Fig. 1C). However, when the SAR was increased to 2.2, 2.42, 2.86, and 3.3 W/kg, the recognition indices were significantly increased (p < 0.05) by 9.45% (n = 10 sham-exposed, n = 11 RF-EMR-exposed), 11.97% (n = 8 sham-exposed, n = 9 RF-EMR-exposed), 13.94% (n = 7 sham-exposed, n = 8 RF-EMR-exposed), and 21.25% (n = 12 sham-exposed, n = 12 RF-EMR-exposed), respectively. Moreover, the RF-EMR-induced increase in recognition memory was a long-term effect. We measured the recognition indices for 8 days after RF-EMR exposure, and showed that recognition memory remained significantly increased compared with controls throughout that period (Fig. 1D), though the increase relative to the sham-exposed mice decreased over time when the original data were normalized to their corresponding control (Fig. 1E).

**Figure 2:**
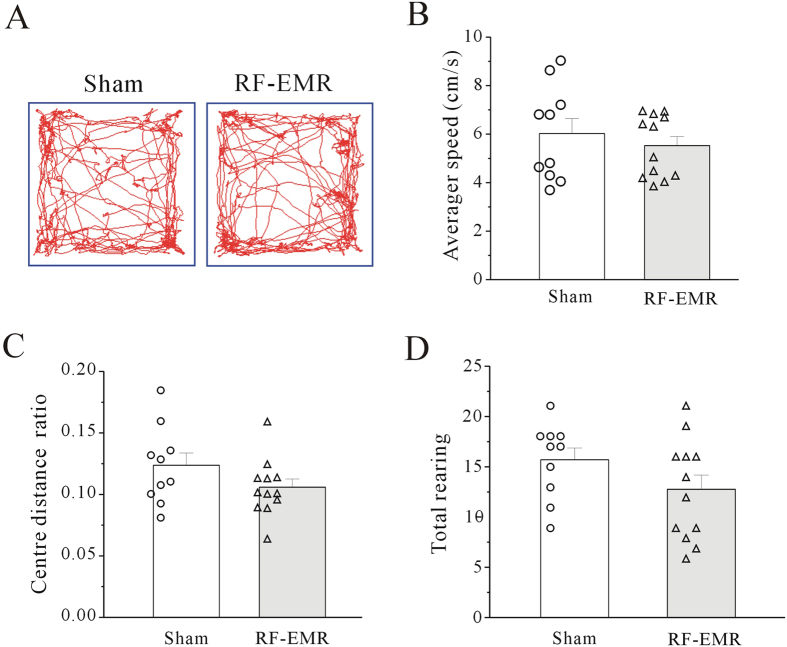
Effect of RF-EMR exposure on locomotor activity in mice evaluated using an open field test. (**A**) Sample traces of sham-exposed and RF-EMR-exposed mice in the open field test. (**B**–**D**) Average speed, central distance ratio, and rearing recorded from sham-exposed and RF-EMR-exposed mice. Data obtained from 22 mice showed no significant differences according to the one-way Kruskal–Wallis test followed by Dunn’s test. The experiment was conducted in each 5-min time period. Hollow circles and triangles indicate original data.

It is possible that the RF-EMR-induced increase in cognition index was due to changes in motor ability or anxiety-like behavior. We assessed these possibilities using open field tests to examine locomotor activity in RF-EMR- (3.3 W/kg) and sham-exposed mice (Fig. 2A). There was no significant difference between the sham-exposed (n = 10) and RF-EMR-exposed groups (n = 12) in terms of average locomotor speed over a 5-min time period (Fig. 2B). Similarly, there was no significant difference in central or total distance ratio or rearing during these 5-min intervals between the sham- and RF-EMR-exposed groups (Fig. 2C and D). These data indicate that a single RF-EMR exposure did not affect murine motor ability.

### RF-EMR exposure increased dendritic-spine densities in hippocampal CA1 pyramidal cells and dendritic-spine length in prefrontal cortical neurons

**Figure 3:**
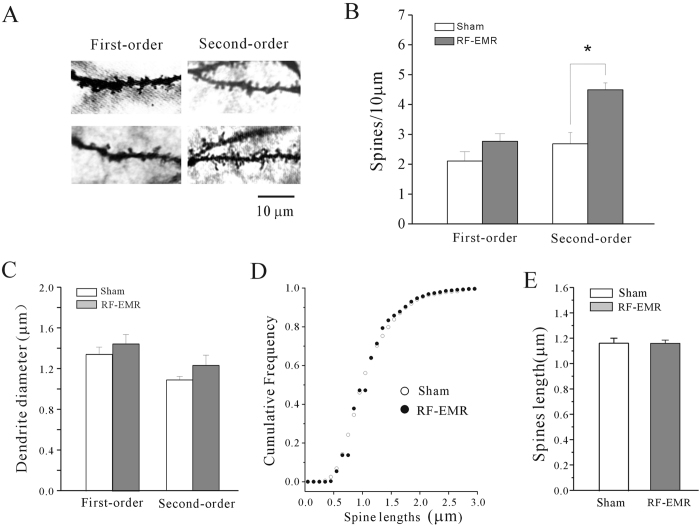
Effect of RF-EMR exposure on hippocampal neuronal morphology observed by Golgi staining. (**A**) Representative images of spine density on first- and second-order dendrites of hippocampus neurons 3 days after 3.3 W/kg RF-EMR exposure for 30 min. Scale bar is 10 μm. (**B**) Statistical analyses of spine density on first- or second-order dendrites of hippocampus neurons. *p < 0.05 for two groups connected by a straight line using a two-sample *t*-test. (**C**) Effect of RF-EMR exposure on dendritic diameter. (**D**) Cumulative frequency distributions of spine length in sham-and RF-EMR-exposed mice compared using Kolmogorov–Smirnov tests. (**E**) Statistical analyses of spine length on first- and second-order dendrites of hippocampus neurons in sham-and RF-EMR-exposed mice.

We recently demonstrated that exposure to extremely low-frequency EMFs (EL-FEFs) (50 Hz) decreased dendritic spine density, which was associated with impaired recognition memory^12^. We therefore investigated the effect of RF-EMR exposure on dendritic-spine densities in hippocampal CA1 pyramidal cells, using Golgi staining. This region was chosen because of evidence linking it to recognition memory^11^,^18^. Spine density was evaluated 3 days after exposure to 3.3 W/kg RF-EMR for 30 min, consistent with the examination of NORT. Spine density was measured on first- and second-order basal dendrites in CA1 pyramidal cells (Fig. 3A), and based on the average value from seven to nine mice in each group. Spine densities in each mouse were evaluated on three to four neurons.

The spine densities on first-order basal dendrites of CA1 pyramidal cells were not significantly affected (n = 21 sham-exposed, n = 17 RF-EMR-exposed, p > 0.05) at 3 days after exposure to 3.3 W/kg RF-EMR for 30 min. However, second-order CA1 pyramidal cell basal dendrites were significantly increased by 69.41%, from 2.68 ± 0.37 per 10 μm in sham-exposed (n = 19) to 4.49 ± 0.23 per 10 μm in RF-EMR-exposed mice (n = 16) (p < 0.05) under the same exposure conditions (Fig. 3B). We also measured dendritic diameter to eliminate possible differences due to diameter variations, and found no significance difference in dendritic diameter between the two groups (Fig. 3C). We also tested for any effect of RF-EMR on spine length in CA1 pyramidal neurons. There was no significant difference in spine lengths between sham- and RF-EMR-exposed mice based on comparison of cumulative frequency distributions using a Kolmogorov–Smirnov test (Fig. 3D and E).

**Figure 4:**
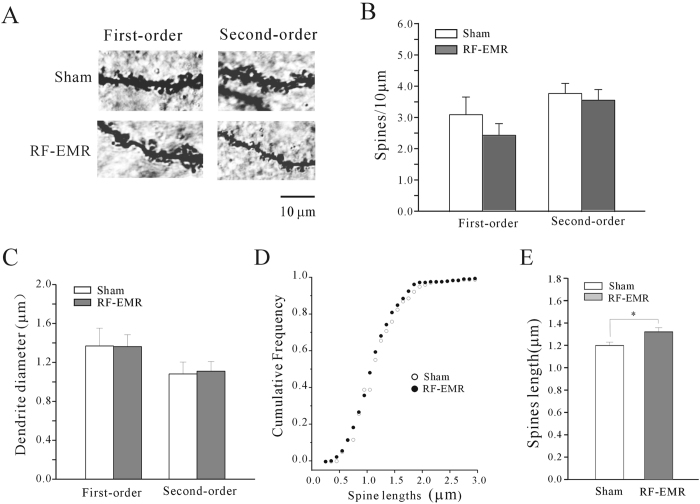
Effect of RF-EMR exposure on prefrontal cortical neuronal morphology observed by Golgi staining. (**A**) Representative images of spine density on first- and second-order dendrites of cortical neurons in sham-exposed mice and at 3 days after 3.3 W/kg RF-EMR-exposure for 30 min. Scale bar is 10 μm. (**B**) Statistical analyses of spine density on first- or second-order dendrites of prefrontal cortical neurons in sham- and RF-EMR-exposed mice. (**C**) Effect of RF-EMR exposure on dendritic diameter. (**D**) Cumulative frequency distributions of spine length in sham- and RF-EMR-exposed mice were compared using Kolmogorov–Smirnov tests. (**E**) Statistical analyses of spine length on first- and second-order dendrites of mPFC neurons in sham- and RF-EMR-exposed mice. *p < 0.05 for two groups connected by a straight line using a two-sample *t*-test.

We observed the effect of RF-EMR exposure on dendritic-spine densities in prefrontal cortex pyramidal cells (Fig. 4A), which have previously been associated with NORT^6^. Spine density was measured on first- and second-order basal dendrites in prefrontal cortical cells, and the average values from five mice in each group were compared. Spine densities within each mouse were evaluated on three to four neurons. In contrast to the results for the hippocampus, there was no significant difference in dendritic-spine densities between the sham- and RF-EMR-exposed mice for either first- (n = 12 sham-exposed, n = 18 RF-EMR-exposed) or second-order basal dendrites (n = 10 sham-exposed, n = 16 RF-EMR-exposed) (Fig. 4B). Similarly, there was no significant increase in dendritic diameter between the two groups (Fig. 4C). However, comparison of cumulative frequency distributions of spine length between sham- and RF-EMR-exposed mice using the Kolmogorov–Smirnov test (Fig. 4D) indicated a slight increase (10.23%) in spine length between the two groups, from 1.19 ± 0.03 in sham-exposed (n = 12) to 1.31 ± 0.03 in RF-EMR-exposed mice (n = 18) (p < 0.05, Fig. 4E).

### RF-EMR exposure enhanced pyramidal neuron excitability in prefrontal cortical and hippocampal slices

RF-EMR exposure has previously been shown to impair synaptic transmission to pyramidal cells in the prefrontal cortex^6^. We therefore determined if single, high-density RF-EMR exposure changed the excitability of neurons in medial prefrontal cortical (mPFC) and hippocampal CA1 pyramidal cells using a whole-cell current-clamp technique. We obtained hippocampal and prefrontal cortical slices from mice after exposure to 3.3 W/kg RF-EMR for 30 min for 3 days, consistent with the protocol used for NORT.

**Figure 5:**
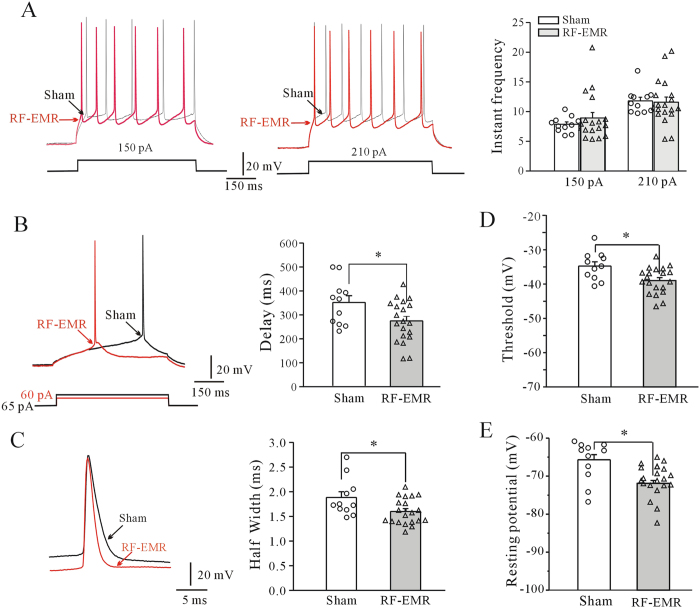
Effect of RF-EMR exposure on action potentials (APs) and membrane potentials in prefrontal cortical neurons. (**A**) Representative current-clamp recording and statistical analyses of AP frequency of cortical neurons in sham- and RF-EMR-exposed mice. (**B**,**C**) Representative sample and statistical analyses showing effect of RF-EMR exposure on delay of first AP elicited by current injection, and half-width of AP in sham- and RF-EMR-exposed mice. (**D**,**E**) Effect of RF-EMR exposure on resting membrane potential and threshold potential in cortical neurons in sham- and RF-EMR-exposed mice. *p < 0.05, for two groups connected by a straight line using a two-sample *t*-test.

Pyramidal neurons in cortical layers III–IV were injected with current to evoke action potential (AP) firing. The injected current density ranged from −30 to 330 pA with a 500 ms duration and increment of 3 pA. Neurons were clamped at a holding potential of −80 mV, which is close to the neuronal resting membrane potential. RF-EMR exposure had no significant effect on AP firing frequency in neurons subjected to 150 or 210 pA (Fig. 5A). However, RF-EMR exposure significantly reduced the delay to elicit an AP from 349 ± 28.32 ms to 273.68 ± 19.30 ms (n = 11 sham-exposed, n = 20 RF-EMR-exposed) (p < 0.05, Fig. 5B), and reduced the threshold from −34.75 ± 1.24 mV to −38.97 ± 0.86 mV (n = 11 sham-exposed, n = 20 RF-EMR-exposed) (p < 0.05, Fig. 5D). The AP half-width was also significantly reduced by 15.01% from 1.88 ± 0.12 ms (sham-exposed, n = 11) to 1.60 ± 0.06 ms (RF-EMR-exposed, n = 20) (p < 0.05, Fig. 5C) in mPFC pyramidal neurons from RF-EMR-exposed mice. In addition, RF-EMR exposure significantly decreased the neuronal resting potential from −65.94 ± 1.62 mV in sham-exposed (n = 11) to −71.15 ± 0.96 mV in RF-EMR-exposed mice (n = 20) (p < 0.05, Fig. 5E).

**Figure 6:**
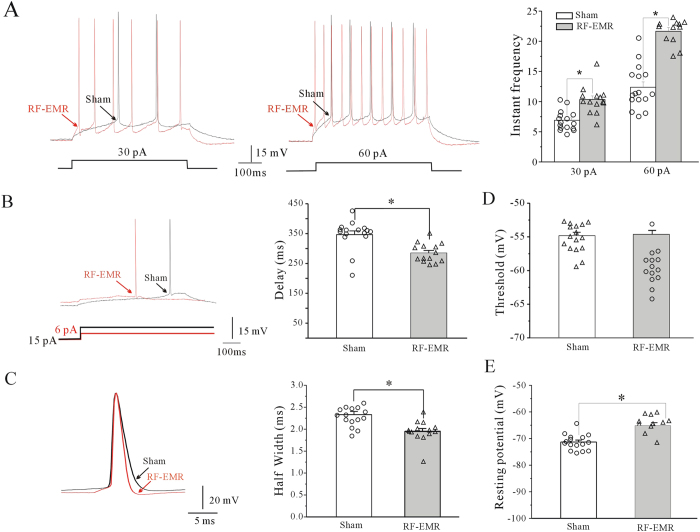
Effect of RF-EMR exposure on action potentials (APs) and membrane potentials in hippocampal CA1 neurons observed by current-clamp recording. (**A**) Representative current-clamp recording and statistical analyses of AP frequency of hippocampal CA1 neurons in sham-exposed mice and 3 days after 3.3 W/kg RF-EMR exposure for 30 min. (**B**,**C**) Representative sample and statistical analyses showing the effect of RF-EMR exposure on delay of the first AP elicited by current injection, and the half-width of AP in sham- and RF-EMR-exposed mice. (**D**,**E**) Effect of RF-EMR exposure on resting membrane potential and threshold potential in hippocampal CA1 neurons in sham- and RF-EMR-exposed mice. *p < 0.05 for two groups connected by a straight line using a two-sample *t*-test.

We also injected pyramidal neurons in hippocampal CA1 with current to evoke AP firing, as described for cortical neurons. The current amplitude required to elicit an AP was smaller in hippocampal CA1 neuron compared with mPFC pyramidal neurons. RF-EMR exposure significantly increased the AP firing frequency by 49.83% (from 6.90 ± 0.41 in sham-exposed [n = 16] to 10.34 ± 0.61 in RF-EMR-exposed [n = 14], p < 0.05) or 75.01% (from 12.37 ± 0.89 in sham-exposed [n = 16] to 21.66 ± 0.60 in RF-EMR-exposed [n = 12], p < 0.05) in neurons injected with 30 pA or 60 pA, respectively (Fig. 6A). Similarly, RF-EMR exposure significantly reduced the delay to elicit an AP from 346.70 ± 12.29 ms in sham-exposed (n = 16) to 284.91 ± 8.61 ms in RF-EMR-exposed mice (n = 14) (p < 0.05, Fig. 6B), and reduced the AP half-width by 16.48% from 2.3 ± 0.07 ms (sham, n = 16) to 1.94 ± 0.07 ms (RF-EMR, n = 14) (p < 0.05, Fig. 6C) in hippocampal neurons. However, RF-EMR exposure did not affect the AP threshold (Fig. 6D). Moreover, RF-EMR exposure significantly depolarized the resting potential from −71.31 ± 0.74 mV (sham, n = 16) to −65.33 ± 1.02 mV (RF-EMR, n = 14) (p < 0.05, Fig. 6E).

Taken together, these data suggest that RF-EMR exposure modified pyramidal neuron excitability in the mPFC and hippocampus in mice.

## Discussion

**Figure 7:**
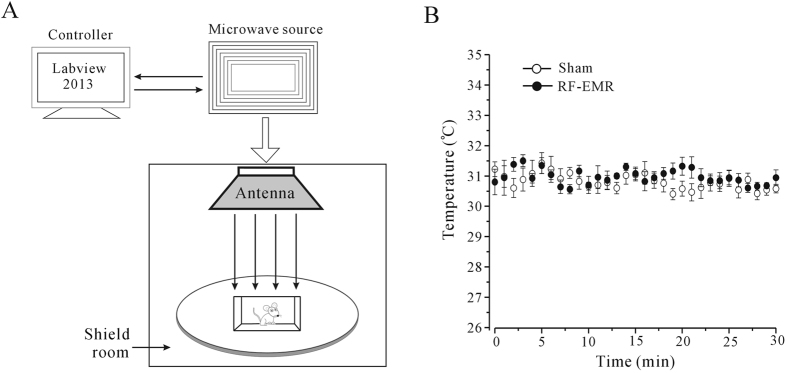
Scheme of RF-EMR-exposure system and its effect on the surface body temperature of mice. (**A**) Schematic diagram of experimental set-up of the RF-EMR-exposure system. (**B**) Time course of changes in surface body temperature obtained from sham- (n = 5) and RF-EMF-exposed mice (n = 5). One-way ANOVA test with Tukey’s post-hoc test indicated no significant differences in surface body temperature between sham-and RF-EMF-exposed groups or among different RF-EMF exposure times.

Given that previous literature has tended to focus on the effects of relatively low-density, long-term RF-EMR exposure, we aimed to determine if more intense and acute RF-EMR exposure would have similar effects. The maximum SAR of RF-EMR (3.3 W/kg for 30 min) used in the current study had no significant effect on surface body temperature (Fig. 7B). This was in accord with the results of Wang *et al*.^17^, who reported no increase in head temperature in mice receiving 10 mW/cm^2^ during exposure to 2.856 GHz microwave radiation for 6 min^17^. However, we did reveal changes in object recognition, dendritic-spine density and length in hippocampal and mPFC neurons, and in neuronal excitability in hippocampal and mPFC in RF-EMR-exposed mice. These results indicate that RF-EMR exposure exerted direct, non-thermally-mediated effects.

In NORT, mice spend more time exploring novel compared with familiar objects, which is taken to indicate memory of the familiar object. RF-EMR exposure increased the time spent exploring novel objects, thus suggesting enhanced recognition memory of the familiar object. Previous studies reported that RF-EMR exposure affected emotionality and stress behavior in rats^19^,^20^, and we therefore determined if the failure in object discrimination might be a function of compromised motor ability or anxiety-related responses using an open field test to measure the center distance and average speed^21^. We found no significant difference in spontaneous locomotor activity between RF-EMR-exposed and control mice, suggesting that RF-EMR did not cause ataxia or motor impairments under the experimental parameters used in this study.

Previous studies of the effects of RF-EMR exposure on learning and memory using the NORT have produced inconsistent findings. Junior *et al*. found that RF-EMR exposure (1.8 GHz) for 25 s every 2 min for 3 consecutive days induced neither anxiety nor impaired working memory in adult (60-day-old) rats. However, stress behaviors, including immobility time and rearing frequency, were significantly increased under these conditions^20^. In comparison, Ntzouni *et al*. found that exposure to different acute and chronic exposure protocols of mobile-phone radiation (SAR value 0.22 W/kg) for 90 min/per day for 17 days significantly affected the consolidation phase of recognition memory in mice^22^. Moreover, Aldad *et al*. demonstrated that fetal exposure to 800–1900 MHz-rated RF radiation led to a deficit in novel object recognition as well as neurophysiological alterations that persisted into adulthood^6^. The results of the current study indicate that an acute and single exposure to RF-EMR (1.8 GHz, 2.2 W/kg) for 30 min is sufficient to increase novel object recognition memory. Given that the radiation component is the key factor in RF-EMR studies, the overall effects must be largely attributable to the related parameters, including frequency, modulation, daily repetition, and peak/average E-field intensity throughout exposure, and variations in these may lead to controversial results. In addition, to the best of our knowledge, this study provides the first evidence for the effects of single exposure to RF-EMR at high density. Notably, we demonstrated that the effects of a high-density, single RF-EMR exposure on increased novel object recognition could last for at least 8 days. This long-term response not only eliminates the possibility of a thermal effect, but also suggests long-term effects on the underlying molecular and cellular mechanisms involved in novel object recognition.

Dendritic spines are the primary site of excitatory input on most principal neurons, and are essential for functional synaptic plasticity and cognition functioning^23^. A study by Xiong *et al*. indicated that exposing rats to 50 Hz ELFEFs for 14 or 28 days decreased spine density in the superficial layers of the medial entorhinal cortex^24^. Similarly, we recently found that chronic exposure to ELFMFs decreased spine density in hippocampal neurons, associated with ELFMF-exposure-induced impairment of novel object recognition memory, and increasing dendritic-spine density by over-expression of hippocampal neuritin with adeno-associated virus reversed novel object recognition impairment^12^. Narayanan *et al*. found that Wistar rats exposed to RF-EMR (900 MHz; peak power density of 146.60 μW/cm^2^) for 1 h/day for 28 consecutive days had significantly reduced dendritic branch points at the 40–60 μm concentric zone, and a progressive decrease in learning abilities measured using the Morris water maze test. They concluded that structural changes in the hippocampus in RF-EMR-exposed rats could be one possible reason for their altered cognitive abilities^25^. Although our results conversely indicated a positive effect of RF-EMR exposure in terms of enhanced novel object recognition and a parallel increase in spine density in hippocampal neurons, the relationship between hippocampal spine density and recognition memory was still retained. We also noted that a high-density single exposure to RF-EMR increased spine length rather than spine density in mPFC neurons. Qin *et al*. showed that a larger percentage of the spines that increased in length were filopodial-type spines, which were unlikely to have formed synaptic contacts^21^, and the number of spines may be more important than spine length in hippocampus-dependent recognition memory^12^. Further studies are required to determine if filopodial-type spines are increased by EMR exposure in this exposure model.

In addition to neuronal morphology, RF-EMR-induced impairments of learning and memory have also been associated with alterations of neural physiology function. For example, 10 mW/cm^2^ microwave radiation significantly reduced spatial memory in rats, assessed by the Morris water maze task performed 6 h after exposure, with changes in population spike amplitudes of long-term potentiation in the hippocampus^17^. Prenatal exposure to 800–1900 MHz cellular phones with a SAR of 1.6 W/kg caused adult impairment in recognition memory measured by NORT, with reduced miniature excitatory postsynaptic currents of pyramidal cells in the prefrontal cortex^6^. The results of the current study demonstrated that RF-EMR exposure significantly increased the frequency of hippocampus neuronal APs. Moreover, RF-EMR exposure depolarized the resting potential and reduced the AP-firing threshold, making it easier for neurons to generate APs and indicating that RF-EMR exposure can significantly enhance hippocampus neuronal excitability. A previous study suggested that the number of spines, and synapse stabilization and maturation were pivotal for excitatory network formation in normal cortical function^26^. Further investigations are needed to determine if the increase in hippocampus neuronal excitability induced by RF-EMR exposure is causally associated with RF-EMR-enhanced spine density.

Recognition memory refers to the ability to judge a previously encountered item as familiar, and depends on the integrity of the medial temporal lobe^27^,^28^. Despite the fact that the anatomical structures involved are mixed, the hippocampus and perirhinal cortex are widely accepted as important and pivotal neuroanatomical substrates for multiple novel object recognition^29^,^30^, while the mPFC has been associated primarily with the object-in-place task, in which animals have to remember which object has been seen and its corresponding location^31^,^32^. Notably, the current study indicated that RF-EMR exposure could significantly enhance the excitability of mPFC neurons by hyperpolarizing the resting potential and reducing the threshold, but without affecting AP frequency. The effects of RF-EMR exposure on neuronal activity differed between hippocampal and cortical neurons, suggesting cell-type-specific or regional variations in susceptibility to RF-EMR. This process occurs simultaneously with an RF-EMR-exposure-induced increase in novel object associative recognition memory. These results, together with those of previous studies^6^, suggest that the mPFC may contribute to RF-EMR-induced enhanced NORT. However, the whole brain was exposed to RF-EMR in our study, and we therefore cannot rule out the possible involvement of other brain regions in the effects of RF-EMR on memory function. Further studies are needed to confirm these results.

In the present study, we determined that a single, acute RF-EMR exposure (3.3 W/kg SAR for 30 min) resulted in enhanced object recognition memory without corresponding changes in spontaneous locomotor activity. We also identified parallel morphological and functional changes in hippocampal and mPFC neurons, though the molecular mechanisms responsible for the effects remain unclear. Lower RF-EMR density and long-term (chronic) exposure-induced cellular/organismal responses have previously been suggested to involve activation of signal transduction pathways, disruption of ion channels, DNA damage, and changes to cellular apoptosis and gene expression^16^,^33^,^34^. We used acute, single, high-density RF-EMR exposure, and showed a fast and long-term positive effect on recognition memory. We therefore speculate that the increased excitatory synaptic activity and spine number were induced by an enzyme or signal transduction pathway, with alteration of neuronal ion channel activity being the most likely option. Finally however, it is worth noting that the European Union and United States have set SAR limits of 2.0 W/kg and 1.6 W/kg, respectively^35^, and the range of 1.98–3.3 W/kg RF-EMR level used in this study was therefore outside the range generally experienced in daily life. Future studies are therefore planned to determine if acute exposure to high-power 1.8 GHz RF-EMR may have therapeutic effects in disorders associated with recognition memory deficits.

## Materials and Methods

### Ethics and animal use statement

This study was conducted in strict accordance to the recommendations of the Guide for the Care and Use of Laboratory Animals of the National Institutes of Health. The protocol was approved by the Committee on the Ethics of Animal Experimentation at Fudan University (permit no. 20090614-001). All surgery was performed under sodium pentobarbital anesthesia (50 mg/kg, intraperitoneal) and all efforts were made to minimize animal suffering.

### Animals

Experimental subjects were 3–4-week-old female C57/LB mice (SLC Co. Ltd., Shanghai, China). The mice were housed in plastic cages at room temperature (23 °C–25 °C) with *ad libitum* access to standard food pellets and water. No specific dietary supplements were provided. The light–dark cycle was set at 12 h (lights on from 8:00 am to 8:00 pm).

### Exposure system and EMF application

The 1.8 GHz electromagnetic irradiation system for live animals included a computer, a microwave signal source, and a two-dimensional movable loading platform. The microwave output from a horn antenna with an adjustable power of 1–200 W. The duration of emission could be set within 0–24 h. The aperture plane of the horn antenna was 21.4 × 16.3 cm^2^. The electric field was measured using an EMF meter (PMM8053B, PMM Costruzioni Electtroniche Centro Misure Radio Electriche S.r.l., Italy) in the exposure groups, which were exposed to emitted powers of 90, 100, 110, 130, and 150 W, respectively. The root mean square values of the electric field at a distance of 1.5 m from the front of the antenna were 138.8, 146.4, 153.5, 166.9, and 179.3 V/m, respectively. Male mice (body weight 30 g) were subjected to magnetic resonance imaging (Magnetom 3 T Trio, Siemens, Germany) with an image resolution of 0.1 × 0.1 × 1.5 mm. Seventeen tissue types were distinguished, including brain, tongue, eyes, esophagus, heart, liver, lung, spleen, stomach, kidney, digestive system, urinary bladder, testis, penis, bone, skin, and muscle. The reference electric constants of the tissues were derived from http://transition.fcc.gov/oet/rfsafety/dielectric.html. The average SAR of whole body and brain were calculated by XFDTD7.2.3. The SARs in brain at the emitted powers were 1.98, 2.2, 2.42, 2.86, and 3.3 W/kg, respectively.

A schematic diagram of the experimental set-up for RF-EMR exposure is presented in Fig. 7A. There were two cages of mice for each experimental group (RF-EMR-exposed and sham-exposed). We simultaneously recorded the surface body temperatures of mice for 30 min using an infrared thermal camera (VcR580C, Infra Tec GmbH, Germany), which could measure mouse body surface temperature with a sensitivity of 0.05 °C in real-time and over long durations. The mice moved freely within the confines of the cage, and the temperatures of various parts of the body therefore differed by approximately 1 °C (29.68 ± 0.35 to 31.7 ± 0.45 °C, n = 10, for sham-exposed and 29.84 ± 0.35 to 32.06 ± 0.24 °C, n = 5, for RF-EMR-exposed). However, exposure to 3.3 W/kg RF-EMF did not significantly increase the surface body temperature of mice (Fig. 7B).

### Open field test

Locomotor activity was evaluated in the control (no RF-EMR exposure) and RF-EMR-exposed groups. The open field apparatus consisted of a clear Plexiglas box (23 × 23 × 35 cm) with a black floor. Mice were placed in the open field in the dark. Activity was detected by a computer-operated tracking system comprising 16 light beams per side (Mobiledatum Information Technology Co., Ltd., Shanghai, China) and recorded continuously in 5-min increments. The total vertical distance moved, center/total distance ratio, and movement speed were measured for each subject (Mobiledatum Information Technology Co., Ltd.).

### NORT

The protocol for NORT was adapted from Bevins and Ennaceur^9^,^36^ and utilized an open field consisting of a clear Plexiglas box (45 × 45 × 45 cm). NORT was conducted in this open field apparatus with 5-min daily trials for 3 consecutive days. On the first day, mice were allowed to explore and habituate to an empty open field box. On the second day, mice were presented with two of the same objects, indicated as A and B. After 24 h, the mice were exposed to two different objects: A and C, with C indicating a novel object. All objects were rinsed with ethanol and allowed to dry between trials and before the first trial. The testing and training sessions were videotaped and analyzed by an experimenter blinded to the treatment group of the animals. Object exploration was defined as each instance in which a mouse’s nose or forelimb touched the object or was oriented towards and within 2 cm of the object. Exploratory activity within the experimental arena was measured over each 5-min trial using a computer timer.

### Golgi staining and image quantification

Golgi staining was performed using an FD Rapid Golgi Stain Kit (FD Neurotechnologies, Baltimore, MD, USA), according to previously described methods^37^. In brief, mouse brains were removed after RF-EMR or sham exposure, placed in the appropriate impregnation solution, and stored in the dark for 14 days at room temperature. The brains were then transferred into a solution containing sucrose and incubated at 4 °C to remove all the residual water from the tissue (2–7 days). Finally, 100 μm thick, semi-horizontal sections including the hippocampus and coronal brain slices (100 μm) containing the prefrontal cortex were obtained using an oscillating tissue slicer (Leica, VT1000, Wetzlar, Germany). The slices were mounted onto gelatin-coated slides and allowed to air dry at room temperature for approximately 2 days, rinsed in distilled water, and incubated for 10 min in a solution containing silver nitrate. Slides were then rinsed again in distilled water before being dehydrated in absolute alcohol, cleared with xylene, and covered with non-acidic synthetic balsam and cover slips.

All image acquisition and analyses were performed by an experimenter who was blinded to the treatment group. When comparing our slices to reference sections^38^, we selected slices obtained from within the anatomical range of −1.5 to 3.0 mm from the bregma for hippocampus and initiation to 0.8 mm for mPFC. The images for dendritic-spine analysis were obtained using a Leica microscope at 1000x magnification. To ensure a homogenous neuronal population, neurons that were selected for analysis had to fulfill the following criteria: (1) the cell body and dendrites were completely impregnated; and (2) the neuron(s) were isolated from the surrounding neurons. We reconstructed intact cell morphology and calculated dendritic length using image analysis software (Neurolucida V9.0, Williston, VT, USA). Dendritic-spine number and length, and dendrite diameter were counted over 20 μm lengths at the beginning of the dendrites of pyramidal neurons in the CA1 region of the hippocampus and in the mPFC. Spine density was expressed as the average number of spines per micron of dendritic length.

### Acute slice preparation

Three- to four-week-old adult mice were deeply anesthetized with pentobarbital sodium before rapid decapitation and removal of the entire brain into ice-cold, oxygenated cutting solution (220 mM sucrose, 3 mM KCl, 5 mM MgCl_2_, 1 mM CaCl_2_, 1.25 mM NaH_2_PO_4_, 26 mM NaHCO_3_, 10 mM glucose, 315 mOsm/l, 95% O_2_/5% CO_2_ and pH 7.3). Coronal slices (200 μm) of the prefrontal cortex (bregma 3.6–2.5 mm) were cut using a vibrating microtome (VT1200S; Leica), and then incubated in artificial cerebral spinal fluid (ACSF) (125 mM NaCl, 2.5 mM KCl, 2.5 mM CaCl_2_, 1.5 mM MgSO_4_, 1 mM NaH_2_PO_4_, 26.2 mM NaHCO_3_, 11 mM glucose, 305 mOsm/L and pH 7.3) for 1 h at 34 °C. Slices were stored at room temperature until experimental use.

For hippocampus slices, entire brains were placed into ice-cold, oxygenated cutting solution (130.05 mM NaCl, 4.96 mM KCl, 0.30 mM MgSO_4_, 11.02 mM MgCl_2_ * 6H_2_O, 20.01 mM HEPES, 1.5 mM CaCl_2_, 22.02 mM NaHCO_3_, 5.50 mM glucose, pH 7.3 adjusted by NaOH, 350–360 mOsm/l, 95% O_2_/5% CO_2_). The brain was then blocked for sectioning. Coronal slices (300–400 μm thick) were cut using a Vibratome (VT1200S, Leica), and incubated in ACSF (109.99 mM NaCl, 2.50 mM KCl, 2.50 mM CaCl_2_, 1.50 mM MgSO_4_, 1 mM KH_2_PO_4_, 26.19 mM NaHCO_3_, 11 mM glucose, 300–310 mOsm/L and pH 7.3) for 1 h at 34 °C. Slices were stored at room temperature until experimental use.

### Whole-cell patch-clamp recording

AP recordings were performed on pyramidal neurons in layers III and IV of cortical slices and CA1 of hippocampal slices in current-clamp mode. Prior to AP recording, ACSF was replaced with a bath solution containing 140 mM NaCl, 2.5 mM KCl, 10 mM HEPES, and 1 mM MgCl_2_ (pH adjusted to 7.4 using NaOH), or 119 mM NaCl, 2.5 mM KCl, 1 mM Na_2_HPO_4_, 26.2 mM NaHCO_3_, 3 mM MgSO_4_, 1 mM CaCl_2_, and 11 mM glucose. The internal pipette solution contained 150 mM K^+^ gluconate, 0.4 mM ethyleneglycolbisaminoethylethertetra-acetate, 8 mM NaCl, 2 mM ATP·Mg, 0.1 mM GTP·Na_3_, and 10 mM N-2-hydroxyethylpiperazine-N”-2-ethanesulfonic acid (HEPES), with pH adjusted to 7.4 by KOH. Resting membrane potential and APs were recorded in the current-clamp mode. The series resistance (Rs) was 20–30 MΩ. Data were discarded if the Rs of the recorded cells varied by >15%. Recordings from cortical and hippocampal neurons were performed at 23 °C–25 °C and 28 °C–30 °C, respectively.

### Statistical analysis

Data were presented as mean ± standard error or as medians. Statistical analyses were performed using GraphPad Prism software (GraphPad Software, Inc., USA). All statistical assessments were two-sided and the corresponding p values were calculated. One-way Kruskal–Wallis tests followed by Dunn’s test were used for multiple comparisons of recognition indices and results of locomotor activity. Two-sample comparisons for normalized recognition indices, dendrite diameters, spine densities and lengths were performed using unpaired two-sample *t*-tests. One-way analysis of variance (ANOVA) was used for multiple comparisons of surface body temperature and different RF-EMR exposure times. All resulting significant differences were analyzed using Tukey’s post-hoc tests.

## Additional Information

**How to cite this article:** Wang, K. *et al*. Effect of 1.8 GHz radiofrequency electromagnetic radiation on novel object associative recognition memory in mice. *Sci. Rep.* **7**, 44521; doi: 10.1038/srep44521 (2017).

**Publisher's note:** Springer Nature remains neutral with regard to jurisdictional claims in published maps and institutional affiliations.
